# Occult *Helicobacter pylori* infection in children: molecular detection and association with gastric dysbiosis

**DOI:** 10.3389/fmicb.2026.1836526

**Published:** 2026-06-15

**Authors:** Qize Li, Mei Long, Cheng Fan, Xiaosong Zhao, Peng Wang, Wenhua Chen, Yan Liu, Junbin Shao, Zhenghong Chen

**Affiliations:** 1 Guizhou Key Laboratory of Microbiome and Infectious Disease Prevention and Control, Guizhou Medical University, Guiyang, Guizhou, China; 2 Department of Pediatric Gastroenterology, Guiyang Maternal and Child Health Care Hospital, Guiyang, Guizhou, China; 3 The Second People’s Hospital of Guiyang City, No. 547, Jinyang South Road, Guanshanhu District, Guiyang, Guizhou, China; 4 Shanghai Zhijiang Biotechnology Co., Ltd., Shanghai, China

**Keywords:** children, chronic inflammation, gastric microbiota, gastric mucosa, occult *H. pylori* infection

## Abstract

Occult *Helicobacter pylori* (Hp) infection, characterized by a low bacterial load that often escapes detection by conventional diagnostic methods, may influence treatment response and prognosis in pediatric patients. In this study we aimed to identify occult *H. pylori* infection in children using molecular techniques and to characterize associated alterations in the gastric microbiota. We enrolled pediatric patients with gastrointestinal disorders who underwent routine clinical diagnostic testing for *Helicobacter pylori* infection, including ^13^C-urea breath test, rapid urease test performed on gastric mucosal biopsy specimens, and/or histopathological examination of gastric tissue. A diagnosis of *H. pylori* infection was established when at least two of these tests yielded concordant positive results. Gastric mucosal samples were further analyzed using *H. pylori*-specific 16S rRNA nested polymerase chain reaction (PCR), glmM quantitative PCR, and next-generation sequencing. Patients who tested negative for *H. pylori* by routine clinical diagnostic assays—but positive by at least one of three molecular methods [glmM quantitative reverse transcription PCR (qRT-PCR), 16S rRNA gene nested PCR, or 16S rRNA gene sequencing]—were classified as having occult *H. pylori* infection. Among 153 enrolled children, 17 were clinically diagnosed with *H. pylori* infection. Of the 136 clinically negative patients, 55 were positive by molecular testing, indicating occult infection. Children with occult infection exhibited chronic gastric mucosal inflammation and demonstrated greater microbial richness and diversity than those with clinically confirmed infection. SparCC analysis revealed a negative correlation between *Akkermansia* abundance and *H. pylori* levels. LEfSe analysis identified enrichment of *Ralstonia* and *Bacteroides* in the occult infection group. Predicted functional profiling—performed using PICRUSt2 and annotated against the Kyoto Encyclopedia of Genes and Genomes (KEGG) Orthology database—revealed significant enrichment of inferred ABC transporter–related and environmental adaptation–associated metabolic pathways in the occult *H. pylori* infection group. These findings indicate that occult *H. pylori* infection was prevalent in our cohort of symptomatic children, frequently undetected by conventional diagnostic methods, and is associated with persistent mucosal inflammation and distinct gastric microbial signatures.

## Introduction

*Helicobacter pylori* is a gram-negative pathogen colonizing the human stomach (Li et al., 2023). As the leading cause of chronic gastritis, *H. pylori* infection can also result in severe gastroduodenal diseases in some patients, including gastric and duodenal peptic ulcers, gastric cancer, and gastric mucosa-associated lymphoid tissue (MALT) lymphoma (Li et al., 2023). Owing to improvements in socioeconomic status and hygiene conditions, *H. pylori* infection rate in children has decreased from 42.2% before 2000 to 34% in 2020 (Malfertheiner et al., 2023). A meta-analysis in China evaluated the prevalence of *H. pylori* infection in children nationwide from 2014 to 2023 and found that the prevalence rate was still 27.5% (Xie et al., 2024). Therefore, early and accurate diagnosis of *H. pylori* infection is essential for enabling precise treatment and preventing the progression of conditions such as atrophic gastritis and gastric cancer.

Currently, *H. pylori* infection is mainly diagnosed using C-13 urea breath test, rapid urease test (RUT), and histopathological examination. Notably, it has been reported that despite using traditional detection procedures, some cases of *H. pylori* infection remain undiagnosed (Ramírez-Lázaro et al., 2020). Multiple independent studies have consistently demonstrated that molecular techniques including nested PCR and 16S rRNA gene sequencing-exhibit significantly higher analytical sensitivity for detecting low-biomass *H. pylori* infections, which commonly evade detection by conventional culture- and histology-based diagnostic methods (Bik et al., 2006; Šeligová et al., 2020). Occult infection is defined as an infection with bacterial density below the detection threshold of conventional diagnostic tests (Ramírez-Lázaro et al., 2015). Early detection and accurate diagnosis of occult *H. pylori* infection in children may facilitate timely intervention, improve treatment outcomes, and reduce the likelihood of missed diagnoses. However, research on this condition in pediatric populations remains limited.

In recent years, gastric microecology has emerged as a novel perspective in the study of *H. pylori* infection. The human gastrointestinal microbiota is frequently considered a functional organ; alterations in its composition can render the host more susceptible to inflammation and disease (Liu et al., 2024). Research shows that the composition and diversity of gastric bacteria differ significantly between patients with *H. pylori* infection and those without it (Hua et al., 2023). In the gastric microbiota of *H. pylori*-infected children, *H. pylori* is the predominant component of the genus *Helicobacter* (Miao et al., 2020). Following *H. pylori* eradication therapy, the richness and composition of the gastric microbiota in these children closely resemble those in uninfected individuals (Miao et al., 2020). However, existing studies on the effect of *H. pylori* infection on gastric microbiota have mainly concentrated on clinically diagnosed positive cases. To date, no published study has specifically characterized the gastric microbiota during the occult phase of *H. pylori* infection in children, nor has any directly compared microbial community profiles between occult and clinical infections within the same pediatric cohort. Given the lack of prior empirical data on this specific clinical phenotype, the present study was designed as an exploratory, hypothesis-generating investigation-rather than a confirmatory, hypothesis-driven trial. Nevertheless, drawing on robust evidence that even low-biomass *H. pylori* colonization can trigger gastric mucosal inflammation (Ramírez-Lázaro et al., 2015), we hypothesized that occult infection would be associated with distinct compositional and functional shifts in the gastric microbiota, alongside persistent low-grade chronic inflammation.

Therefore, The primary objectives of this study were to (1) apply validated molecular assays including glmM quantitative PCR, 16S rRNA gene nested PCR, and 16S rRNA gene sequencing - to detect occult *H. pylori* infection in symptomatic children; (2) determine the prevalence of occult infection within this pediatric cohort; and (3) comparatively characterize gastric microbiota composition across three clinically defined groups: children with molecularly confirmed occult *H. pylori* infection, those with conventional clinical diagnosis of *H. pylori* infection, and *H. pylori*-negative controls with intact gastric mucosa.

## Materials and methods

### Study cohort

This study involved pediatric patients under 18 years of age who presented with recurrent abdominal pain, abdominal distension, vomiting, hematemesis, and other related symptoms at Guiyang Maternal and Child Health Hospital and Guiyang Second People’s Hospital between July 2024 and February 2025. All participants underwent gastroscopy examination. Exclusion criteria included: use of bismuth-containing agents, proton pump inhibitors, antibiotics, H_2_-receptor antagonists, or nonsteroidal anti-inflammatory drugs within 4 weeks prior to enrollment; and a gastric mucosal DNA concentration of less than 1 ng/μL in the extracted sample. This study was conducted in accordance with the principles of the Declaration of Helsinki and approved by the Medical Ethics Committee of Guiyang Maternal and Child Health Hospital (Approval No.: fybjy-2024-102) and the Medical Ethics Committee of Guiyang Second People’s Hospital (Approval No.: jyyy-2024-xm-12). Written informed consent was obtained from the legal guardians of all participating children.

### Classification of *H. pylori* infection status

Based on the aforementioned detection methods for *H. pylori*, the study cohort was categorized into three groups: the clinically diagnosed *H. pylori* group, occult *H. pylori* infection group, and *H. pylori-*negative group.

Children in the clinically diagnosed *H. pylori* group were required to meet at least one of the following criteria: (1) positive results in both the rapid urease test and histopathological examination; (2) positive results in both the urea breath test and rapid urease test; or (3) during an episode of peptic ulcer bleeding, positive results in both gastric mucosal tissue staining and histopathological examination (Subspecialty Group of Gastroenterology, the Society of Pediatrics, Chinese Medical Association et al., 2023).

Occult *H. pylori* infection was defined as fulfilling all of the following criteria: (i) negativity as defined by the aforementioned clinical diagnostic criteria (i.e., failure to meet the threshold for a clinically diagnosed infection); and (ii) molecular positivity in at least one of three orthogonal, analytically validated assays-namely, glmM-targeted quantitative reverse transcription PCR (qRT-PCR), *H. pylori*-specific 16S rRNA nested PCR followed by Sanger sequencing and BLAST-based taxonomic confirmation, or detection of *H. pylori*-associated amplicon sequence variants (ASVs) in 16S rRNA gene sequencing data. A positive result in any single molecular assay was sufficient to classify an individual as having occult infection; concordant positivity across multiple assays was not required.

For ethical considerations, healthy children were not included as a control group in this study; instead, individuals in the *H. pylori*-negative group served as a relative control cohort.

#### C13-urea breath test

Patients who had fasted for at least 6 h were administered a capsule containing 100 mg of C13-labeled urea. Breath samples were collected at the baseline (prior to administration) and 30 min after administration (T30 minus T0). The ratio of exhaled ^13^C to ^12^C was measured in parts per thousand (‰). The final result is expressed as the difference between these two measurements, known as the delta over baseline (DOB). A cutoff value of 4‰ DOB was established according to the manufacturer’s instructions (Shenzhen Zhonghe Haidewei Biotechnology Co., Ltd., Shenzhen, China), with results exceeding this threshold considered positive.

#### RUT

Endoscopic examinations were performed at Guiyang Maternal and Child Health Hospital and Guiyang Second People’s Hospital. After gastroscopy, gastric mucosal tissue specimens were collected and immediately placed at the center of the yellow reagent pad of an RUT strip (Zhuhai Kedi Technology Development Co., Ltd. Zhuhai, China). The backing paper was then securely resealed to ensure an airtight seal between the test strip and backing paper. Color changes were monitored within 15 min; development of a red color on the test strip was interpreted as positive for urease activity.

#### Histopathology

A subset of patients consented to undergo gastric tissue sampling for histopathological examination after the completion of gastroscopy. Histopathological evaluation was independently conducted by two board-certified pathologists blinded to the results of all other clinical and laboratory investigations. In cases of discordance, the pathologists jointly reviewed the histologic slides and reached a consensus through structured discussion. Although formal inter-rater agreement statistics (e.g., Cohen’s kappa) were not calculated, the consensus-based adjudication process was implemented to mitigate subjective interpretation bias. All biopsy specimens designated for histological analysis were fixed in 10% buffered formalin and subsequently embedded in paraffin. Consecutive sections were subjected to hematoxylin–eosin and May–Giemsa staining. Samples showing the presence of *H. pylori* on the pathological slides were classified as positive. Neutrophil activity, inflammation, atrophy, and intestinal metaplasia were graded into four categories (0: normal; 1: mild; 2: moderate; 3: severe) according to the updated Sydney system (Homan et al., 2024).

#### qRT-PCR

Two pieces of antral tissue were collected from each pediatric patient for molecular testing. The gastric mucosa samples were homogenized prior to DNA extraction. DNA was extracted using a DNA extraction kit (Z-ME-0044; Shanghai Liferiver Company) in accordance with the manufacturer’s instructions. Throughout the DNA extraction process, ultrapure water was used as a negative control in place of the sample solution to prevent potential false-positive PCR results.

*H. pylori* (glmM) and its associated toxins (VacA/CagA) were detected using a nucleic acid detection kit (SD-0318-02; Shanghai Liferiver Company). The total volume of the PCR mixture was 40 μL, including 4 μL of DNA template, 35 μL of *H. pylori* nucleic acid qRT-PCR detection mixture, and 0.4 μL of Taq enzyme. The PCR amplification procedure comprised the following steps: pre-denaturation at 94 °C for 2 min; denaturation at 93 °C for 15 s; followed by annealing at 60 °C for 60 s, repeated for a total of 40 cycles. Each run incorporated a positive control (*H. pylori* strain 26695) and a negative control (water).

#### Nested PCR

The *H. pylori* 16S rRNA gene was detected using a nested PCR approach for gene amplification. The outer primers for the first round were *HeliN* 5’-AAGAACCTTACCTAGGCTTGACATTG-3’ and *HeliN* 5’-CCGTGGGCAG TAGCCAATT-3’. For the second round, the inner primers used were *Hpup* 5’-TGAGAGAATCCGCTAGAAATAGTGG-3’ and *Hpdown* 5’-TAGCATCCT GACTTAAGGCAAACA-3’ (Šeligová et al., 2020). To reduce the risk of contamination, a negative control was included after each sample in three repeated experiments. The total volume of the reaction mixture for the first round was 25 μL, including 2 μL of DNA template, 1 μL each of upstream and downstream primers, and 12.5 μL of 2 × Hieff^®^ Robust PCR Master Mix, with ddH_2_O added to obtain a final volume of 25 μL. The total volume of the reaction mixture for the second round was also 25 μL, comprising 0.5 μL of DNA template (from the first round), along with the same volumes of reagents as in the first round, including 1 μL each for both upstream and downstream primers. The amplification was performed in a thermal cycler under the conditions reported previously (Homan et al., 2024). *H. pylori* strain ATCC26695 served as a positive control throughout this process. Subsequently, the PCR products were electrophoresed on a 1.5% agarose gel. Samples with positive results were sent to Shanghai Sangon Biotechnology Co., Ltd. for further processing.

#### Second-generation high-throughput sequencing

The V3-V4 region of the bacterial 16S rRNA gene was amplified using universal primers *341F* (5’-CCTAYGGGRBGCASCAG-3’) and *806R* (5’-GGACTACNNGGGTATCTAAT-3’). The total volume of the PCR mixture was 15 μL, which included Phusion High-Fidelity PCR Master Mix, forward and reverse primers at a concentration of 0.2 μM each, and 10 ng of template DNA. The thermal cycling conditions included an initial denaturation step at 98 °C for 1 min, followed by 30 cycles of denaturation at 98 °C for 10 s, annealing at 50 °C for 30 s, and extension at 72 °C for 30 s; the final extension was performed at 72 °C for an additional 5 min. To control for potential contamination in low-biomass samples, ultrapure water was used as a negative control during both DNA extraction and PCR amplification. No amplifiable products were detected in any of the negative controls. The resulting PCR products were purified using magnetic beads and subsequently pooled in equal volumes based on their concentrations. After thorough mixing, the PCR products were analyzed to recover the target bands. A library was constructed, and its quality was assessed using Qubit and qPCR prior to sequencing.

#### Sequence processing and ASV inference

Raw paired-end reads—targeting the 16S rRNA gene V3-V4 region and generated on the Illumina NovaSeq platform by Novogene Co., Ltd.—were demultiplexed and primer-trimmed using cutadapt (implemented within QIIME2). Quality filtering was performed with fastp (v0.23.1), which excluded reads containing more than five ambiguous (N) bases, required a minimum Phred quality score of ≥ 19, and permitted no more than 15% low-quality bases (quality < Q15) per read. To account for NovaSeq’s compressed quality encoding scheme—where quality scores are binned into discrete values (2: no-call; 12: < Q15; 23: ∼Q20; 37: > Q30)—DADA2 (Callahan et al., 2016) was applied with forward and reverse read truncation at 240 and 200 bp, respectively; trimming from the 5’ end commenced at the first base with a quality score < 10 (trunc_q = 10); and reads with more than two expected errors were discarded (max_ee = 2). Paired-end reads were merged using the QIIME2 default settings, requiring a minimum overlap of 12 bp. Chimeric sequences were identified and removed using UCHIME (*de novo* mode) against the SILVA v138.1 reference database. The resulting amplicon sequence variant (ASV) table was rarefied to a uniform depth of 100,000 sequences per sample—the minimum sequencing depth across all samples. Taxonomic assignment was conducted using QIIME2’s scikit-learn–based classifier trained on the SILVA v138.1 99% OTU representative set. ASVs remaining unclassified at the genus level were further refined via BLASTN alignment against the NCBI 16S rRNA database (release date: March 2025). The abundance table representing these sequences within the samples is referred to as the feature table. Based on this feature table, we assessed whether *H. pylori* sequences were present; if detected, samples were classified as positive.

### Bioinformatic analysis

The ASV table, representative sequences, and phylogenetic tree were imported into the QIIME 2 analysis platform.^1^ Within this framework, α- and β-diversity analyses were performed on three groups: children with clinically diagnosed *H. pylori* infection, those with occult *H. pylori* infection, and *H. pylori-*negative individuals. Differences in microbial community structure among the groups were assessed for statistical significance using Adonis and ANOSIM. Statistical comparisons further revealed the taxa exhibiting significant abundance shifts across groups and determined the patterns of taxon enrichment associated with each group. Next, the microbiota of children with mild (A2_1) and moderate (A2_2) chronic gastritis within the occult *H. pylori* infection group were compared to identify microbial communities that may synergize with *H. pylori* to promote inflammation.

### Functional prediction analysis

To investigate the potential physiological effects of occult *H. pylori* infection, functional profiling of the gastric microbiota was conducted using PICRUSt2 (v2.6.2) with default parameters (Douglas et al., 2020). Briefly, ASV representative sequences were placed into a reference phylogeny using EPA-NG and GAPPA; gene family abundances were inferred via hidden-state prediction implemented in the castor R package; and the predicted metagenomic content was subsequently collapsed to KEGG Ortholog (KO) abundances. Pathway enrichment analyses were then performed to compare functional profiles across experimental groups.

Importantly, these functional predictions represent computational inferences derived from 16S rRNA gene sequencing data and reference genomes, rather than direct experimental measurements of microbial functional activity (e.g., meta-transcriptomics or metabolomics). Consequently, the inferred functional profiles should be interpreted as exploratory and hypothesis-generating-rather than definitive.

### Statistical analysis

In the clinical dataset, count data are expressed as ratio or proportion, and continuous variables are reported as mean ± standard deviation. Group comparisons were performed using *t*-tests for two groups and analysis of variance or chi-square tests for multiple groups, as appropriate. Statistical significance was set at *P* < 0.05. For non-parametric analyses, the Kruskal -Wallis test and all pairwise Wilcoxon rank-sum tests were conducted with an adjusted α level of 0.05. Pairwise comparisons of alpha diversity were conducted using the Wilcoxon rank-sum test, with *p*-values adjusted for multiple testing via the Benjamini-Hochberg false discovery rate (FDR) procedure (reported as *q*-values). Co-occurrence and co-exclusion patterns among taxa were assessed at the genus level using the SparCC algorithm. For LEfSe analysis, the initial Kruskal-Wallis and pairwise Wilcoxon tests were performed without additional multiple testing correction, as the method’s multi-step filtering (including linear discriminant analysis (LDA) thresholding) reduces false discovery risk. In LDA, effect sizes with an absolute LDA score of > 3.0 and a *P*-value of < 0.05 were considered statistically significant.

No a priori sample size calculation or formal statistical power analysis was conducted prior to patient enrollment, as this study was explicitly designed as an exploratory investigation rather than a hypothesis-driven, confirmatory trial.

## Results

### Positive rates of various *H. pylori* detection methods and experimental grouping

**FIGURE 1:**
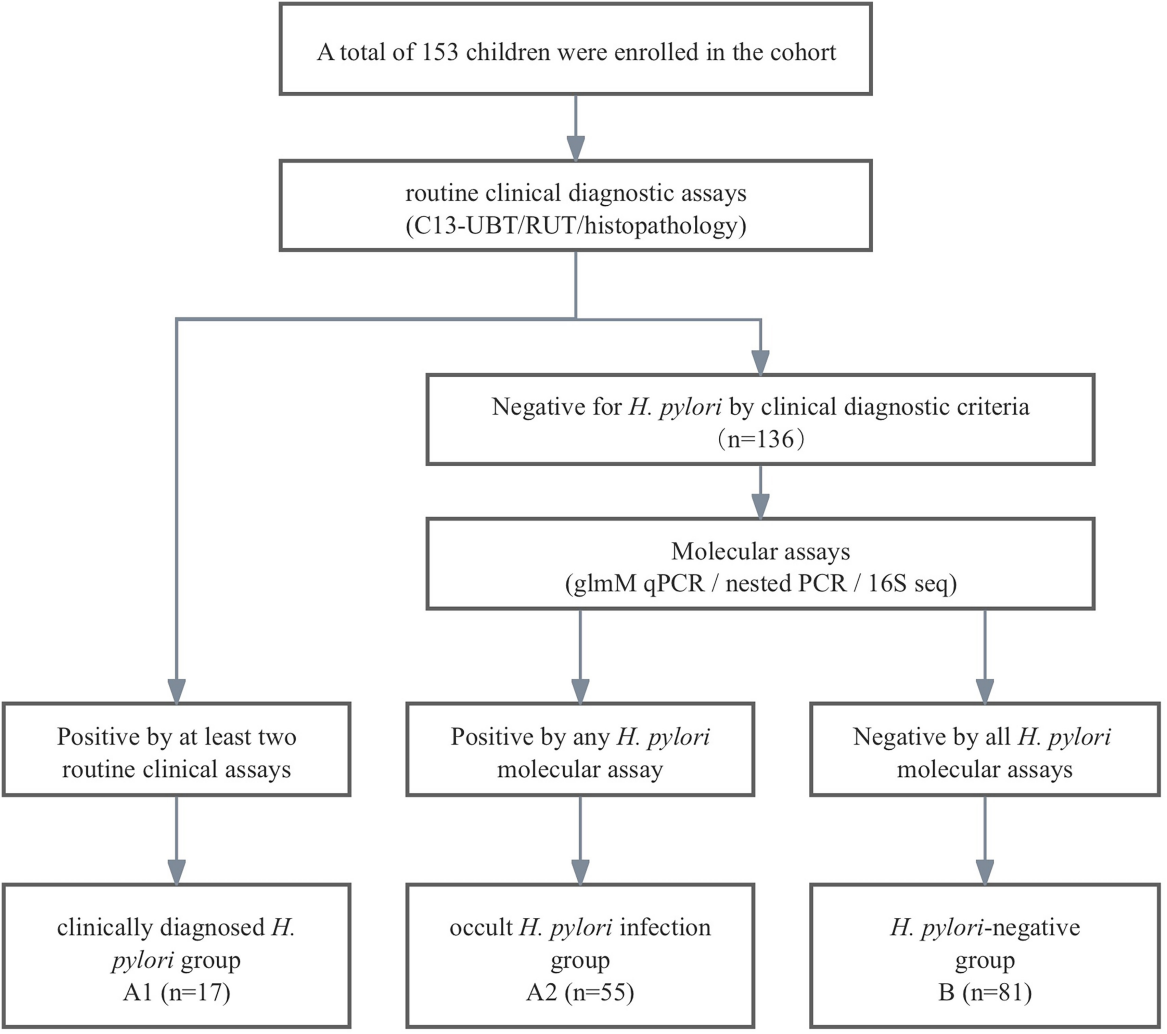
Flowchart of patient enrollment and group classification. Of the 168 enrolled children, 153 were retained after DNA quality control. Patients with ≥ 2 positive clinical tests were assigned to the clinically diagnosed *H. pylori* infection group (A1; *n* = 17). The remaining 136 underwent molecular testing; those with a positive result in at least one assay were assigned to the occult *H. pylori* infection group (A2; *n* = 55), and those with negative results across all assays were assigned to the *H. pylori*-negative group (B; *n* = 81).

Among the 153 gastric mucosa samples collected from patients. Microbiome sequencing was performed on the V3–V4 region of the 16S rRNA gene for all 153 samples. Concurrently, qRT-PCR and nested PCR assays were conducted on these samples. Among 153 samples, 20 (13.07%) tested positive in the C^13^ urea breath test and 19 tested positive (12.42%) in the RUT. In the molecular analyses, 22 cases (14.38%) tested positive in glmM qRT-PCR, 14 (9.15%) in cagA qRT-PCR, 14 (9.15%) in vacA qRT-PCR, 44 (28.76%) in 16S rRNA nested PCR, and 42 (27.45%) in 16S rRNA high-throughput sequencing (Table 1). To illustrate the extent of overlap among the three molecular methods, a Venn diagram is shown in Supplementary Figure 1. Based on the integrated results from the C^13^ urea breath test, gastric mucosa RUT, histological examination, qRT-PCR, and 16S rRNA gene sequencing, the study population was classified into the following three groups: Group A1 (*n* = 17), Group A2 (*n* = 55), and Group B (*n* = 81) (the diagnostic flowchart is presented in Figure 1).

**TABLE 1:** Detection rate of *H. pylori*.

Detection type	Positive for *H. pylori* (n (%))			
	Total	A1 (*n* = 17)	A2 (*n* = 55)	B (*n* = 81)
C13	20 (13.07%)	14 (82.35%)	4 (7.27%)	2 (2.47%)
RUT	19 (12.42%)	13 (76.47%)	2 (3.64%)	4 (4.94%)
glmM qRT-PCR	22 (14.38%)	14 (82.35%)	8 (14.55%)	0 (0.00%)
cagA qRT-PCR	14 (9.15%)	10 (71.43%)	4 (7.27%)	0 (0.00%)
vacA qRT-PCR	14 (9.15%)	9 (52.94%)	5 (9.09%)	0 (0.00%)
16S nPCR	44 (28.76%)	10 (71.43%)	34 (61.82%)	0 (0.00%)
16S rRNA Sequencing	42 (27.45%)	13 (76.47%)	29 (52.73%)	0 (0.00%)

Group A1, clinical diagnosed *H. pylori* infection group; Group A2, occult *H. pylori* infection group; Group B, *H. pylori*-negative group.

### Clinical information

The clinical characteristics of the patients, including age, sex, clinical symptoms, and endoscopic findings, are summarized in Table 2. No statistically significant differences were observed among the three groups with respect to age, sex, or clinical symptoms (*P* > 0.05). The proportion of children with clinically diagnosed *H. pylori* infection who exhibited nodular gastritis and duodenal ulcers during endoscopy was significantly higher than that of children with occult *H. pylori* infection and *H. pylori*-negative children (*P* < 0.001). In contrast, the prevalence of superficial gastritis was lower in children with clinically diagnosed *H. pylori* infection than that in *H. pylori*-negative children (*P* = 0.038). No significant differences in endoscopic findings were observed between the occult *H. pylori* infection and *H. pylori*-negative groups (*P* > 0.05).

**TABLE 2:** Clinical information of the participants.

Clinical information	A1 (*n* = 17)	A2 (*n* = 55)	B (*n* = 81)	*P*
Mean age ± SD (years)	10.28 ± 2.47	9.56 ± 3.09	9.12 ± 2.75	0.286, 0.368, 0.133, 0.384
Sex, n (%)				
Male	11 (64.71%)	32 (58.18%)	45 (55.56%)	0.780, 0.632, 0.488, 0.762
Symptom, n (%)				
Abdominal pain	16 (94.12%)	51 (92.73%)	71 (87.65%)	0.562, 1.000, 0.683, 0.401
Acid reflux symptom	1 (5.88%)	8 (14.55%)	7 (8.64%)	0.439, 0.678, 1.000, 0.281
Nausea	2 (11.76%)	8 (14.55%)	17 (20.99%)	0.499, 1.000, 0.513, 0.341
Vomiting	8 (47.06%)	20 (36.36%)	21 (25.93%)	0.163, 0.429, 0.083, 0.193
Hematemesis	0 (0.00%)	5 (9.09%)	2 (2.47%)	0.122, 0.331, 1.000, 0.119
Others	0 (0.00%)	1 (1.82%)	3 (3.70%)	0.615, 1.000, 1.000, 0.647
Gastroscopy n (%)				
Esophagitis	1 (5.88%)	1 (1.82%)	1 (1.23%)	0.452, 0.419, 0.318, 1.000
Superficial gastritis	8 (47.06%)	33 (60.00%)	61 (75.31%)	0.034, 0.407, 0.038, 0.058
Erosive gastritis	5 (29.41%)	20 (36.36%)	29 (35.80%)	0.863, 0.773, 0.781, 0.947
Nodular gastritis	7 (41.18%)	1 (1.82%)	1 (1.23%)	< 0.001, <0.001, <0.001, 1.000
Reflux gastritis	1 (5.88%)	6 (10.91%)	9 (11.11%)	0.807, 1.000, 1.000, 0.971
Gastric ulcer	1 (5.88%)	1 (1.82%)	6 (7.41%)	0.353, 0.419, 1.000, 0.241
Duodenitis	3 (17.65%)	21 (38.18%)	32 (39.51%)	0.225, 0.148, 0.102, 0.876
Duodenal ulcer	5 (29.41%)	3 (5.45%)	1 (1.23%)	< 0.001, 0.015, <0.001, 0.303

Group A1, clinically diagnosed *H. pylori* infection group; Group A2, occult *H. pylori* infection group; Group B, *H. pylori*-negative group. *P*-values are presented for the following comparisons: A1 vs. A2 vs. B; A1 vs. A2; A1 vs. B; and A2 vs. B. Additional symptoms include abdominal distension and early satiety.

### Histological findings

A subset of participants in this study were subjected to histopathological examination (Group A1: *n* = 14, Group A2: *n* = 36, Group B: *n* = 47) to assess gastric mucosal damage. No *H. pylori* cells were detected in the histopathological analyses of either the occult *H. pylori* infection group or the *H. pylori*-negative group. According to the updated Sydney system, the clinically diagnosed *H. pylori* infection group showed significantly higher activity and chronic inflammation scores than those of the other two groups (*P* < 0.001). Although the degree of chronic inflammation in the occult *H. pylori* infection group was greater than that in the *H. pylori*-negative group, this difference was not significant (*P* > 0.05). Lymphoid follicle formation was observed in the lamina propria of all three groups; however, the incidence of lymphoid follicle formation was markedly higher in the group clinically diagnosed with *H. pylori* infection compared to that of the other two groups. Atrophy and intestinal metaplasia scores across all groups were zero (Table 3).

**TABLE 3:** Comparison of updated Sydney System scores among Groups A1, A2, and B.

Findings	A1 (*n* = 14)	A2 (*n* = 36)	B (*n* = 47)	*P*
Detection rate of *H. pylori* [n (%)]	10 (71.43%)	0 (0.00%)	0 (0.00%)	< 0.001, <0.001, <0.001, 1.000
Acute activity [n (%)]				< 0.001, <0.001, <0.001, 0.253
0	5 (35.71%)	35 (97.22%)	47 (100.00%)	
1	8 (57.14%)	1 (2.78%)	0 (0.00%)	
2	1 (7.14%)	0 (0.00%)	0 (0.00%)	
3	0 (0.00%)	0 (0.00%)	0 (0.00%)	
Chronic inflammation [n (%)]				< 0.001, <0.001, <0.001, 0.158
0	0 (0.00%)	0 (0.00%)	1 (2.13%)	
1	5 (35.71%)	32 (88.89%)	44 (93.62%)	
2	9 (64.29%)	4 (11.11%)	2 (4.25%)	
3	0 (0.00%)	0 (0.00%)	0 (0.00%)	
Lymphatic follicles [n (%)]	5 (35.71%)	2 (5.56%)	1 (2.13%)	< 0.001, 0.006, <0.001, 0.410

Group A1, clinically diagnosed *H. pylori* infection group; Group A2, occult *H. pylori* infection group; Group B, *H. pylori*-negative group. *P*-values are presented for the following comparisons: A1 vs. A2 vs. B; A1 vs. A2; A1 vs. B; and A2 vs. B.

### Effect of occult H. pylori infection on gastric microecology

**FIGURE 2:**
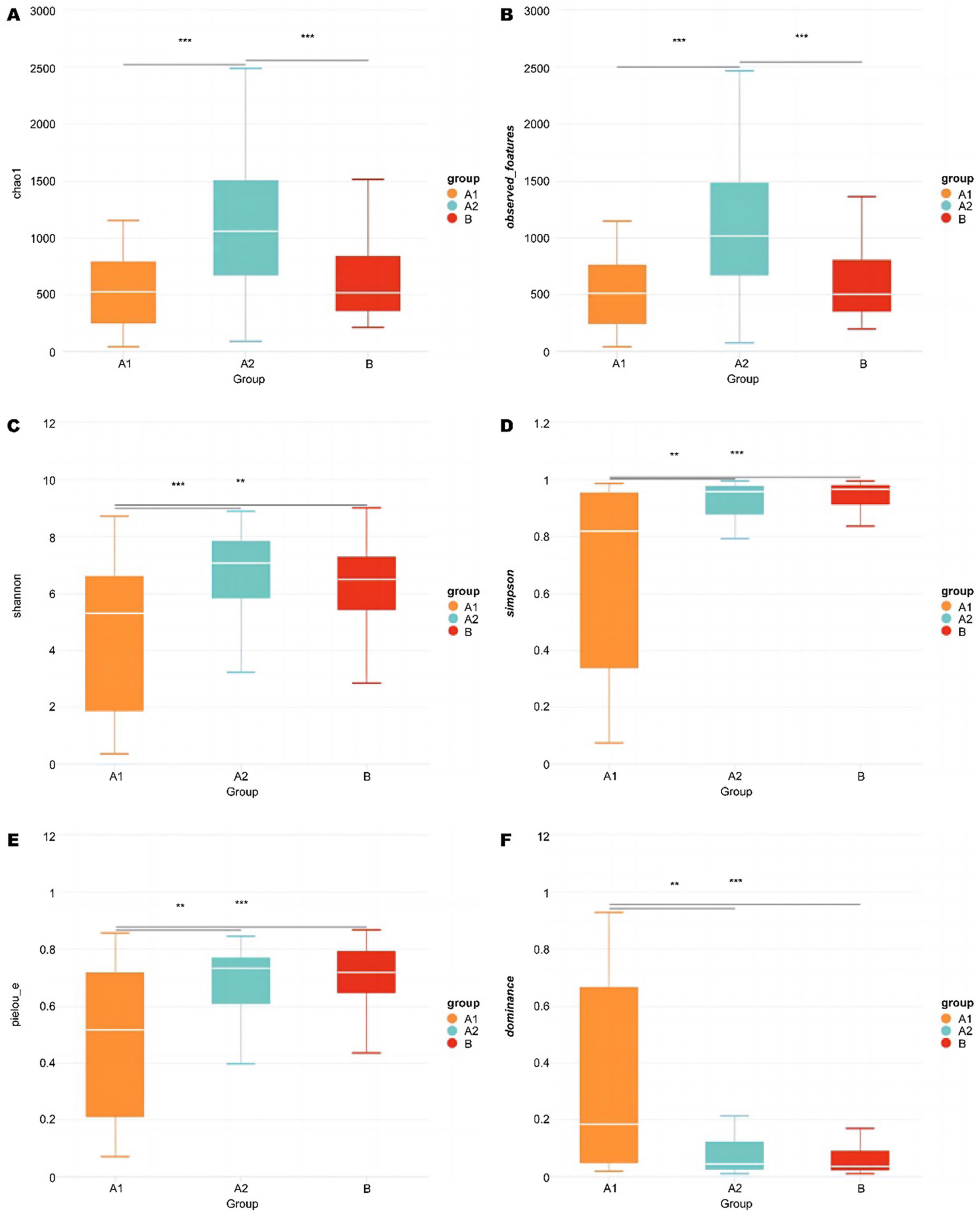
Alpha diversity indices and statistical comparisons among the A1, A2, and B groups. **(A)** Chao1 index across the three groups. **(B)** Observed_features index across the three groups. **(C)** Shannon index across the three groups. **(D)** Simpson index across the three groups. **(E)** Pielou_e evenness index across the three groups. **(F)** Dominance index across the three groups. Higher bacterial richness and diversity were observed in the gastric mucosa of children with occult *H. pylori* infection. In the clinically diagnosed *H. pylori* infection group, species evenness was significantly reduced compared with that in both the occult *H. pylori* infection and *H. pylori*-negative groups. Adjusted q-values are reported: Chao1: A1 vs. A2, *q* = 4.29e-05; A1 vs. B, *q* = 4.00e-04; A2 vs. B, *q* = 0.0682. Observed_features: A1 vs. A2, *q* = 5.04e-05; A1 vs. B, *q* = 5.12e-04; A2 vs. B, *q* = 0.0796. Shannon: A1 vs. A2, *q* = 4.48e-04; A1 vs. B, *q* = 6.28e-03; A2 vs. B, *q* = 0.2083. Simpson: A1 vs. A2, *q* = 6.56e-04; A1 vs. B, *q* = 7.41e-03; A2 vs. B, *q* = 0.3417. Pielou_e: A1 vs. A2, *q* = 9.04e-04; A1 vs. B, *q* = 9.85e-03; A2 vs. B, *q* = 0.2759. Dominance: A1 vs. A2, *q* = 6.56e-04; A1 vs. B, *q* = 7.41e-03; A2 vs. B, *q* = 0.3417 Group A1: clinical diagnosed *H. pylori* infection group; Group A2: occult *H. pylori* infection group; Group B: *H. pylori*-negative group. **P < 0.01; ***P < 0.001.

Alpha diversity was used to assess the microbial community diversity within samples, reflecting both species richness and evenness. The Chao1 and observed_features metrics in the occult *H. pylori* infection group were significantly higher than those in both the clinically diagnosed *H. pylori* infection and *H. pylori*-negative groups (*P* < 0.001), indicating a greater number of detectable bacterial taxa in the gastric mucosa during occult infection. Furthermore, the Simpson index, Shannon diversity index, and Pielou_e evenness values were significantly lower in the clinically diagnosed *H. pylori* infection group than in both the occult *H. pylori* infection and *H. pylori*-negative groups (*P* < 0.01). Conversely, community dominance was significantly higher in the clinically diagnosed *H. pylori* infection group than that in either of the other two groups (*P* < 0.01). These results demonstrate that microbial communities in individuals with clinically diagnosed *H. pylori* infections exhibit markedly reduced species evenness compared with those in individuals with occult infections or *H. pylori*-negative controls (Figure 2).

**FIGURE 3:**
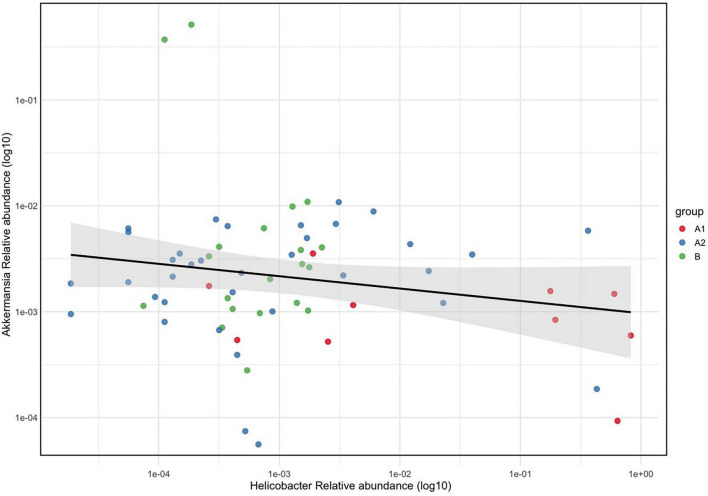
PCoA based on Bray-Curtis distances of gastric microbiota among groups. ADONIS analysis indicated significant differences in β-diversity among the clinically diagnosed *H. pylori* group, occult *H. pylori* group, and *H. pylori*-negative group (*P* < 0.001), with the occult *H. pylori* group exhibiting a more dispersed clustering pattern. Group A1: clinical diagnosed *H. pylori* infection group; Group A2: occult *H. pylori* infection group; Group B: *H. pylori*-negative group.

Beta diversity reflects differences in microbial community structure across samples. We performed principal coordinates analysis based on the weighted UniFrac distance to visualize bacterial community clustering based on abundance. The Bray–Curtis dissimilarity matrix was used to evaluate differences in β-diversity among the three groups. Distinct clustering patterns were observed for each group, and ADONIS analysis of the Bray–Curtis distance matrix confirmed significant differences in microbial composition among the groups (*R*^2^ = 0.046, *P* = 0.001). As shown in Figure 3, the clinically diagnosed *H. pylori* infection group showed a distinct clustering pattern compared to that of both the occult *H. pylori* infection group (*R*^2^ = 0.044, *P* = 0.002) and the *H. pylori*-negative group (*R*^2^ = 0.026, *P* = 0.001). Furthermore, a significant difference in microbial composition was also detected between the occult *H. pylori* infection and *H. pylori*-negative groups (*R*^2^ = 0.034, *P* = 0.001).

### Differences in bacterial compositions

**FIGURE 4:**
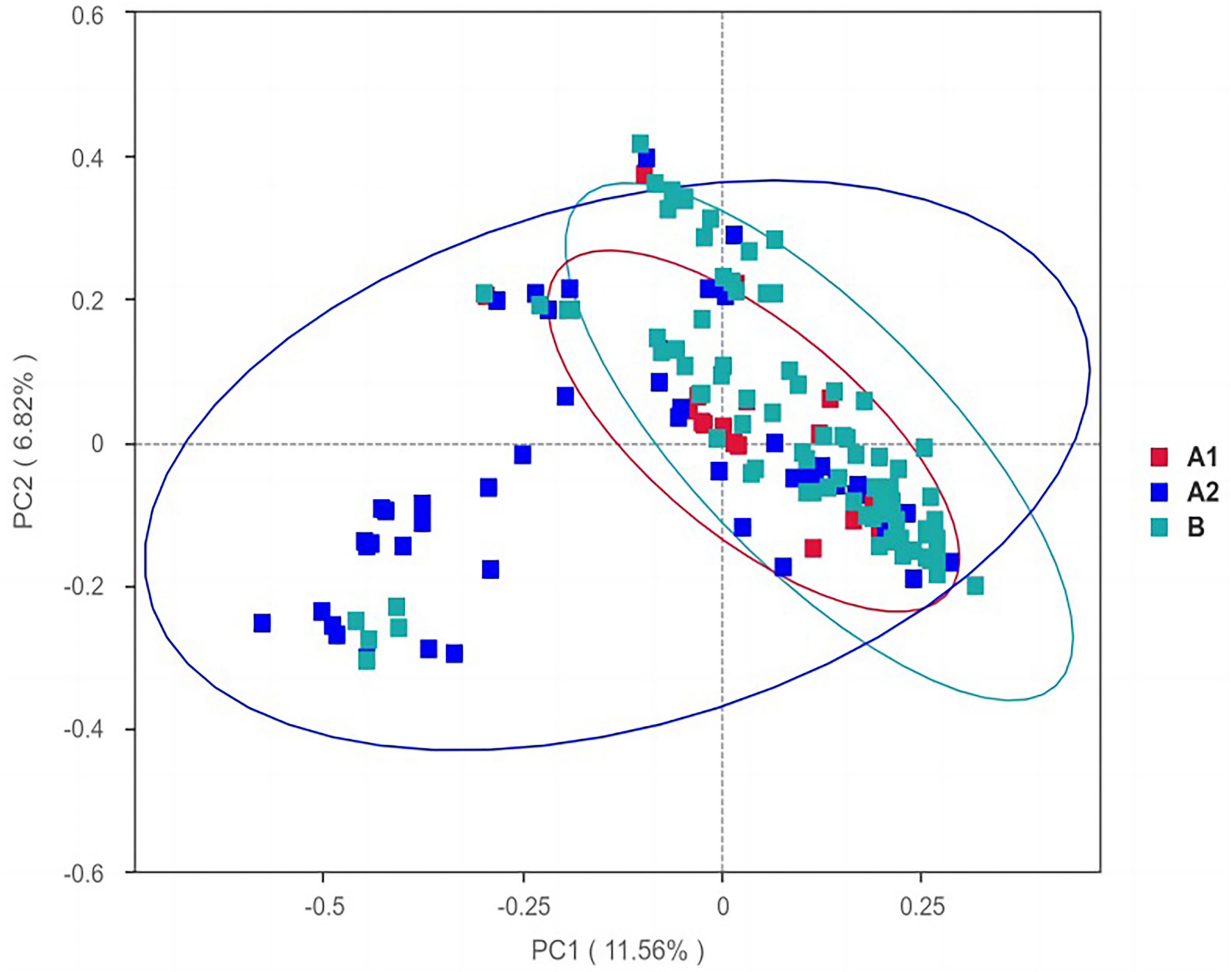
SparCC network plot. A significant negative correlation was observed between the relative abundances of *Akkermansia* and *Helicobacter* in gastric mucosa-associated microbiota (MAM) communities across subjects (*R* = -0.36, *P* = 0.031). Group A1: clinical diagnosed *H. pylori* infection group; Group A2: occult *H. pylori* infection group; Group B: *H. pylori*-negative group.

Based on 16S rRNA gene sequencing, the gastric mucosal microbiome is predominantly composed of *Proteobacteria*, *Campylobacterota*, *Firmicutes*, *Cyanobacteria*, *Actinobacteria*, *Verrucomicrobiota*, *Bacteroidota*, *Aenigmarchaeota*, *Fusobacteriota*, and *Patescibacteria*. Among these phyla, *Proteobacteria* is the most dominant (average relative abundance > 30%). The overall composition and relative abundances of microbial communities across groups are presented in Supplementary Figures 2A,B. In the clinically diagnosed *H. pylori* infection group, the average relative abundance of *Helicobacter* reached 28.54%. In contrast, it was only 1.77% in the occult *H. pylori* infection group and 0.53% in the *H. pylori*-negative group. At the genus level, restricted to taxa with an average relative abundance exceeding 1.0% in at least one group, significant intergroup differences were observed. Individuals with occult *H. pylori* infection showed significantly higher relative abundance of *Ralstonia* (*P* = 0.01) and *Bacteroides* (*P* = 0.001) but lower abundance of *Haemophilus* than the *H. pylori-*negative group (*P* < 0.01). The occult infection group showed significantly higher levels of *Ralstonia* (*P* = 0.01), *Bacteroides* (*P* = 0.001), *Akkermansia* (*P* = 0.001), and *Faecalibacterium* (*P* = 0.001), but significantly lower levels of *Helicobacter* than the clinically diagnosed *H. pylori* infection group (*P* = 0.006). To further explore these microbial interactions, we applied the SparCC algorithm to infer the co-occurrence and co-exclusion patterns at the genus level between *Helicobacter* and taxa with relative abundance > 0.1%. The analysis revealed a significant negative correlation between *Akkermansia* and *Helicobacter* (*R* = -0.36, *P* = 0.031; Figure 4).

**FIGURE 5:**
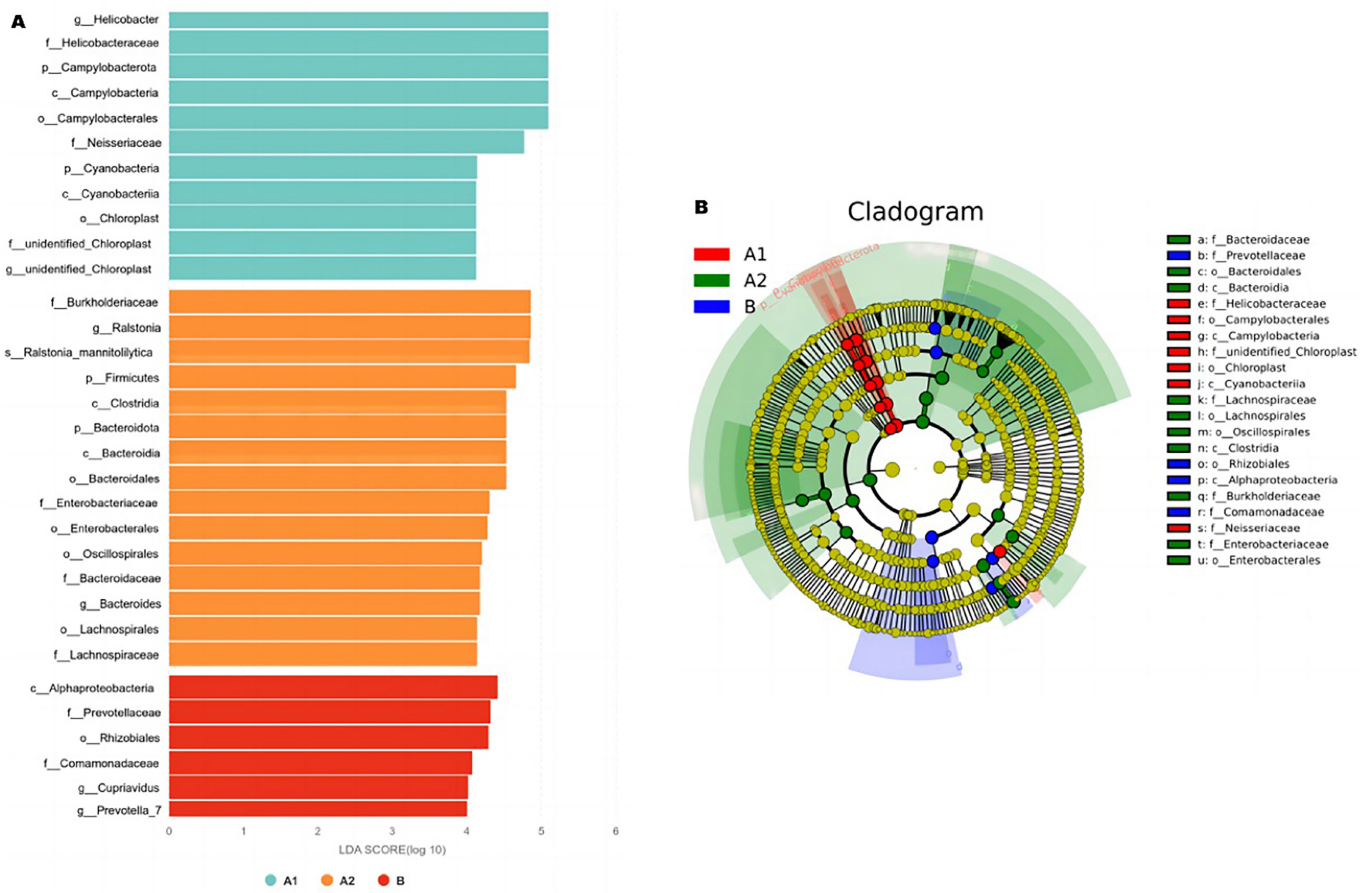
Identification of differentially abundant bacteria using LEfSe analysis. **(A)** Histogram of LDA scores showing bacterial taxa with significant differences among Groups A1, A2, and B. **(B)** Cladogram illustrating the taxonomic distribution of discriminant features across Groups A1, A2, and B. *Ralstonia* and *Bacteroides* were enriched in the occult *H. pylori* infection group, whereas *Helicobacter* was enriched in the clinically diagnosed *H. pylori* infection group. LEfSe analysis was conducted using an LDA score threshold of > 3.0 and a significance level of *P* < 0.05 (Kruskal -Wallis test followed by pairwise Wilcoxon rank-sum tests for multiple comparisons). Group sample sizes: A1 = 17, A2 = 55, B = 81. Group A1: clinical diagnosed *H. pylori* infection group; Group A2: occult *H. pylori* infection group; Group B: *H. pylori*-negative group.

To identify the key bacterial taxa associated with occult *H. pylori* infection, we performed biomarker screening using Linear discriminant analysis Effect Size (LefSe) analysis. In total, 32 differentially abundant taxonomic units, including 6 genera, were identified across the 3 groups (Figure 5A). The taxonomic distribution of these biomarkers is presented in a cladogram (Figure 5B). The LefSe analysis (LDA > 3.0, *P* < 0.05) revealed enrichment of *Ralstonia* and *Bacteroides* in the occult *H. pylori* infection group, *Helicobacter* and *unidentified_chloroplast* in the clinically diagnosed *H. pylori* infection group, and *Prevotella_7* and *Cupriavidus* in the *H. pylori*-negative group.

### Bacteria associated with chronic inflammation

Histological changes associated with *H. pylori* infection may be influenced by the gastric microbiota. To further explore the bacterial taxa associated with the severity of chronic inflammation in children with occult *H. pylori* infection, we stratified these patients into two groups based on histopathological findings: grade 1 and grade 2 chronic inflammation. Subsequently, LefSe analysis was performed to identify potential microbial biomarkers. The results revealed that *Leptotrichiaceae* (family), *Leptotrichia* (genus), *Prevotella_7* (genus), and *Prevotella_melaninogenica* (species) were enriched in the grade 1 chronic inflammation group, whereas *Chloroflexi* (phylum), *Ktedonobacteria* (class), *Chloroflexia* (class), *Streptomycetales* (order), *Sutterellaceae* (family), *Streptomycetaceae* (family), *Acidothermaceae* (family), *Sutterella* (genus), *Streptomyces* (genus), and *Acidothermus* (genus) were enriched in the grade 2 chronic inflammation group (Supplementary Figures 3A,B).

### Functional characteristics of gastric microbiota associated with occult H. pylori infection

**FIGURE 6:**
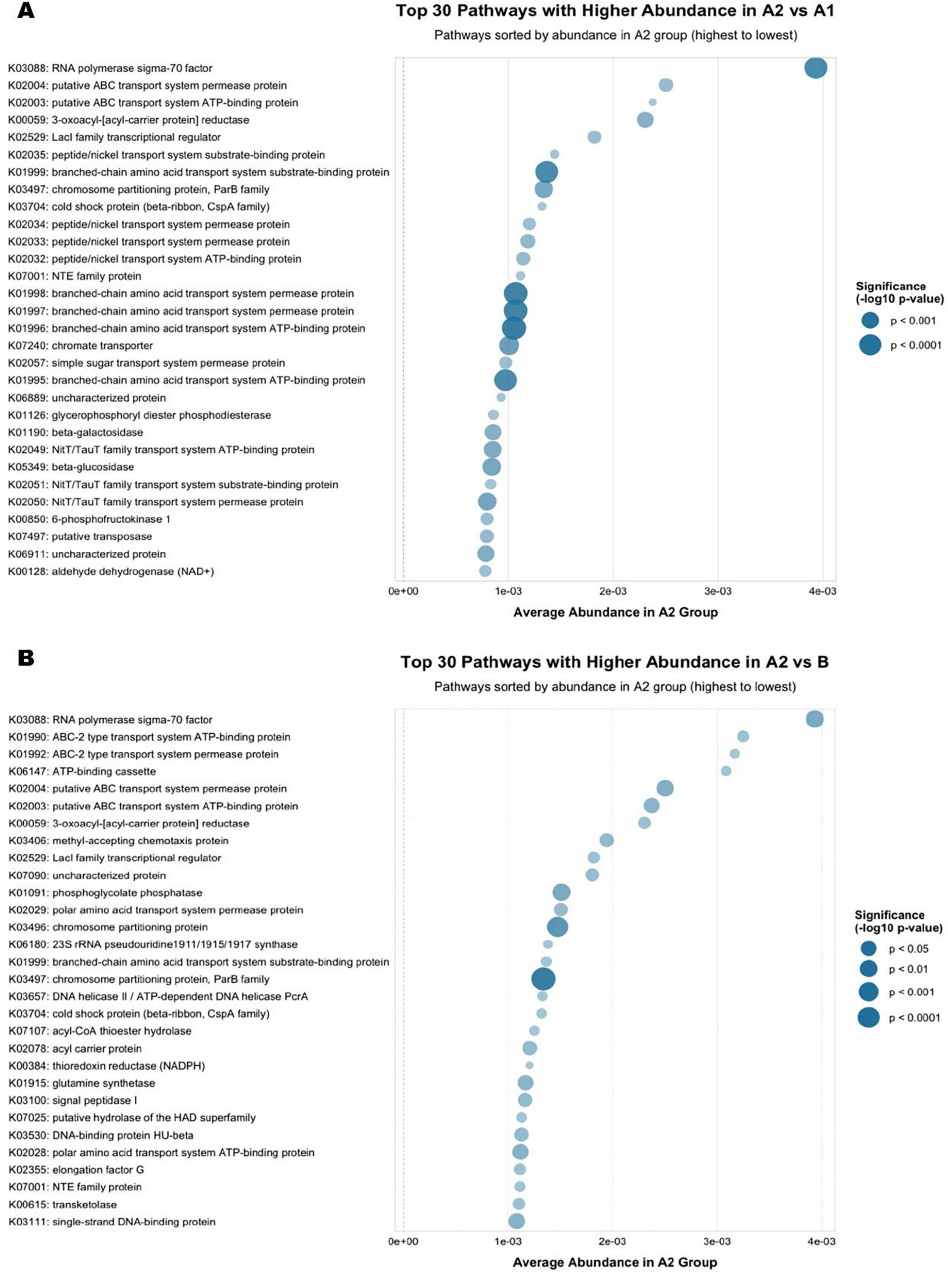
Functional characteristics of the gastric microbiota in Group A2. **(A)** Compared with the clinically diagnosed *H. pylori* infection group, the occult *H. pylori* infection group showed enrichment in pathways related to nutrient uptake and competitive fitness, particularly the ABC transporter system. Genes associated with environmental adaptation and stress response, such as cold shock protein (K03704), σ70 factor (K03088), and chromosome partitioning protein ParB (K03497), were significantly upregulated. This finding suggests active environmental adaptation and continuous nutrient assimilation by the microbial community. Moreover, owing to the low abundance of *H. pylori*, other beneficial microbes retain a pronounced fatty acid-production capacity, as indicated by the high expression of K00059, a key gene in fatty acid biosynthesis. **(B)** Compared with the *H. pylori*-negative group, the occult *H. pylori* infection group exhibited elevated expression of genes involved in DNA replication and cell division, including ParB, DNA helicase, single-stranded DNA binding protein, and elongation factor G. This finding indicates that the microbial community is in an active state of growth and proliferation. Additionally, enhanced expression of σ70 factor, chemotaxis proteins, and proteins of multiple amino acid and nutrient transport systems was observed. Cold shock protein (CspA), DNA-binding protein HU-beta, and thioredoxin reductase are also highly expressed. These findings suggest that the microbiota in the occult *H. pylori* infection group actively acquires nutrients while mounting robust responses to environmental stresses such as temperature shifts, oxidative stress, and nutrient limitation. Collectively, the findings indicate ongoing adaptation to a host microenvironment wherein *H. pylori* persists at low levels under suppression. However, this adaptive state may be associated with sustained low-grade inflammatory stimuli. Group A1, clinical diagnosed *H. pylori* infection group; Group A2, occult *H. pylori* infection group; Group B, *H. pylori*-negative group.

The contribution of the gastric microbiota to pathological states is mediated by its functional potential. We used PICRUSt2 to infer the microbial functional profiles from 16S rRNA gene sequence data. The cluster analysis revealed that the KEGG Orthology (KO) compositions of the three groups clustered in distinct quadrants, indicating differences in bacterial gene functions with partial overlap (Supplementary Figure 4). Therefore, we further analyzed KO-level differences among the occult *H. pylori* infection, clinically diagnosed *H. pylori* infection, and *H. pylori*-negative groups. Compared with the clinically diagnosed *H. pylori* group, the occult *H. pylori* infection group was enriched in genes associated with branched-chain amino acid transport (K01999, K01996–K01998), peptide/nickel transport (K02035, K02032–K02034), other ABC transporters (K02004, K02003), cold shock proteins (K03704), σ70 factors (K03088), chromosome partitioning protein ParB (K03497), and 3-oxoacyl-ACP reductase (K00059, a key enzyme in fatty acid biosynthesis). These enriched pathways suggest an active metabolic state, efficient nutrient uptake, high microbial diversity, strong competitive ability, and enhanced ecosystem stability in the occult infection group. The microbiota appears to be relatively stable, capable of responding appropriately to minor environmental fluctuations under low-grade inflammation, while maintaining active growth, genetic fidelity, and structural and functional integrity. Collectively, these features indicate that the gastric microbiota in the occult *H. pylori* infection group is more metabolically active, stable, diverse, and competitive than that in the clinically diagnosed group, reflecting an ongoing adaptive response to the host environment (Figure 6A). Compared with the *H. pylori*-negative group, the occult *H. pylori* infection group showed enrichment of genes involved in genetic information processing and cell proliferation (K03497, K03496, K03657, K03111, K02355), environmental adaptation and stress response (K03088, K03406, K03704, K03530, K00384), and multiple ABC transporters (K01990, K01992, K02004, K02003, K01999, K02029). This finding indicates sustained high metabolic and proliferative activities, with active reconstruction of a complex and competitive microbial community. Concurrently, the microbiota remains in a “stress-responsive” or “competitive” state, suggesting that occult *H. pylori* infection persists in a dynamically balanced microenvironment where *H. pylori* colonization is suppressed but still exerts biological influence (Figure 6B).

## Discussion

In this study, we aimed to evaluate the prevalence of occult *H. pylori* infection in children by analyzing gastric mucosal specimens, characterizing the gastric microbiota in infected individuals, and elucidating the effect of occult *H. pylori* infection on gastric mucosal tissue and intragastric microecology. Overall, 35.94% of children tested positive for *H. pylori* in molecular biological assays, whereas conventional diagnostic tests, including the C13 urea breath test and RUT, failed to detect *H. pylori*. Similar discrepancies between molecular detection and conventional diagnostics have been reported. For example, one study analyzing 104 gastric biopsy samples from pediatric patients reported that PCR identified 44 cases (42.3%) as *H. pylori* positive, whereas histological examination confirmed colonization in only 39 cases (37.5%) (Bogiel et al., 2022). Collectively, these findings suggest that a substantial proportion of children may harbor *H. pylori* during childhood without being diagnosed.

To enhance interpretability, results are presented and discussed in two complementary layers: (1) molecular detection of *H. pylori* DNA via glmM qPCR, nested PCR, and 16S rRNA gene sequencing; and (2) comprehensive gastric microbiota profiling-including alpha and beta diversity, taxonomic composition, co-occurrence network patterns, and predicted functional pathways.

The marked discordance in positivity rates among molecular assays observed in the occult infection group warrants discussion. Consistent with prior studies (Šeligová et al., 2020), nested PCR demonstrated substantially higher sensitivity than single-round qPCR, attributable to its two-step amplification strategy-which enhances detection of ultra-low *H. pylori* DNA copy numbers. Moreover, the *glmM* gene is present as a single copy in the *H. pylori* genome, whereas the 16S rRNA gene exists in multiple copies; consequently, assays targeting 16S rRNA inherently possess greater analytical sensitivity. In addition, quantitative reverse transcription PCR (qRT-PCR) has a relatively high limit of quantification and may produce false-negative results when bacterial load falls below its linear dynamic range. Furthermore, *H. pylori’*s tendency toward focal gastric colonization may introduce sampling bias in RUT and histopathology, as a single biopsy specimen may fail to capture patchily distributed organisms. In this study, the clinical symptoms or endoscopic findings did not differ significantly between children with occult *H. pylori* infection and those with *H. pylori*-negative test results. This finding suggests that occult *H. pylori* infection is not the sole driver of upper gastrointestinal symptoms or gastric mucosal inflammation in children. Furthermore, it hampers early detection of *H. pylori* infection by clinicians. Therefore, using molecular detection methods to assess *H. pylori* infection and identifying the coexisting pathogenic bacteria may be a more effective diagnostic strategy. Notably, although the proportion of moderate chronic gastritis observed in histopathological specimens from children with occult *H. pylori* infection was higher than that in *H. pylori*-negative children, this difference did not reach statistical significance. Nevertheless, this trend suggests that occult *H. pylori* infection may be associated with increased gastric mucosal inflammation in pediatric patients—a hypothesis that warrants validation in larger, adequately powered cohorts.

In this study, we observed that according to the α-diversity index, the species evenness of the gastric microbiota in patients with occult *H. pylori* infection did not differ significantly from that in *H. pylori*-negative individuals. However, overall diversity was markedly higher in the former group than in the latter. This finding suggests that although new microbial species may emerge during occult *H. pylori* infection, the relative abundance of originally dominant taxa remains largely unchanged. Previous studies have reported that after colonization, *H. pylori* can alter the gastric microbiota by releasing virulence factors, modulating gastric acidity, inducing host immune responses, and competing with resident microbes for resources (Tao et al., 2020). Most studies indicate that both species richness and diversity of the gastric microbiota are lower in *H. pylori-*positive children than in their *H. pylori*-negative counterparts (Miao et al., 2020; Hua et al., 2023). However, our study did not include healthy children as a control group, which may explain the lack of a significant difference in species richness between patients with clinically diagnosed *H. pylori* infection and *H. pylori*-negative cases within the cohort. Notably, significantly lower microbial evenness was observed in patients with clinically diagnosed *H. pylori* infection than in their *H. pylori*-negative counterparts; the β-diversity analyses further revealed distinct compositional differences among the groups (Sugihartono et al., 2022).

Consistent with previous study findings, *H. pylori* infection is associated with an increased relative abundance of Spirochaetes members and reduced relative abundances of Actinobacteria, Bacteroidetes, and Firmicutes members (Liu et al., 2022). In our study, the relative abundance of *Helicobacter* at the genus level was significantly higher in both the clinically diagnosed *H. pylori* infection group and the occult *H. pylori* infection group than that in the *H. pylori-*negative group.

In addition to *Helicobacter*, we observed enrichment of the genera *Ralstonia* and *Bacteroides* in patients with occult *H. pylori* infection. Previous studies have shown that non-*Helicobacter* bacterial taxa that are enriched in *H. pylori*-positive individuals include *Ralstonia, Nocardiopsis, Rhizobium intermedium, Rhizobium leguminosarum*, and *Labrys* (Hua et al., 2023). Notably, 1 year after *H. pylori* eradication, increased *Ralstonia* abundance has been detected in the gastric mucosa of patients with persistent inflammation, suggesting a potential role of this genus in sustaining chronic gastric mucosal inflammation (Sung et al., 2020). *Bacteroides* species are a prevalent commensal found in biofilm communities in patients with inflammatory bowel disease (Dejea et al., 2014). Studies in Mongolian gerbils with long-term *H. pylori* infection have demonstrated that successful *H. pylori* colonization leads to significantly increased *Bacteroides* abundance in the gastric mucosa, indicating that *H. pylori* creates a microenvironment favorable for *Bacteroides* expansion (Yin et al., 2011). Furthermore, enterotoxigenic *Bacteroides fragilis* has been shown to degrade the mucus layer, compromise epithelial barrier function, and exert genotoxic effects on host cells (Li et al., 2017). Thus, excessive proliferation of *Bacteroides* may impair epithelial integrity and contribute to cellular damage. Collectively, enrichment of *Ralstonia* and *Bacteroides* during occult *H. pylori* infection highlights their potential pathogenic contributions to microbial dysbiosis, facilitation of *H. pylori* persistence, and exacerbation of gastric inflammation.

In addition to microbiota that cooperate with *H. pylori* in promoting gastric mucosal inflammation, children with occult *H. pylori* infection may harbor microbial taxa with inhibitory effects against *H. pylori*. This study revealed a negative correlation between *Akkermansia* and *Helicobacter*, suggesting a potential role for *Akkermansia* in suppressing *H. pylori* colonization. Previous studies have also confirmed this inverse association between *Helicobacter* and *Akkermansia* spp. (He et al., 2022). Moreover, mouse models of colorectal cancer have revealed significant enrichment of mucin-degrading microbial features associated with *H. pylori* infection, particularly *Akkermansia* spp. and *Ruminococcus* spp. (Ralser et al., 2023). Thus, *Akkermansia* is hypothesized to inhibit *H. pylori* growth by degrading the mucus layer and contributing to mucosal pathogen clearance.

Our analysis of chronic inflammation in children with occult *H. pylori* infection reveals that *Sutterella* and *Streptomyces* are significantly enriched in cases of moderate chronic inflammation. *Sutterella*, a microaerophilic gram-negative bacterium, has been extensively documented for its pro-inflammatory properties in the gastrointestinal tract and central nervous system (Kaakoush, 2020; Davoody et al., 2025). Notably, *Streptomyces* exhibits a positive association with gastric cancer development (Ai et al., 2023). *H. pylori* infection is the primary risk factor for gastric cancer; chronic colonization of the gastric mucosa by *H. pylori* can cause progressive atrophic gastritis and intestinal metaplasia over time (Smyth et al., 2020). Therefore, although our data do not establish a direct causal relationship, persistent chronic inflammation and microbial dysbiosis—both potentially attributable to occult *H. pylori* infection—may jointly contribute to an elevated long-term risk of gastric cancer development in adulthood. Nevertheless, this hypothesis warrants rigorous validation through well-designed longitudinal studies with extended follow-up periods. In conclusion, varying levels of *H. pylori* colonization alter the gastric microenvironment and influence the gastric microbiota composition. Further research is needed to elucidate the functional roles of specific microbial taxa, which may be critical for preventing and managing *H. pylori* infections.

Furthermore, we demonstrated that the differences in metabolic pathways between the occult *H. pylori* infection group and clinically diagnosed *H. pylori* infection group suggest enhanced colonization capacity through improved nickel transport and environmental adaptation. For instance, enrichment of the cytoplasmic extracellular stress response protein RNA polymerase σ70 factor in the gastric microbiota of children with occult *H. pylori* infection supports upregulated stress adaptation in these individuals (Sharma et al., 2009). Hypothetical ABC transporter permease proteins, which hydrolyze ATP to energize transmembrane transport and facilitate nutrient uptake, are also enriched (Davidson et al., 2008). Peptide/nickel transport system permease proteins actively import nickel ions, providing essential cofactors for urease synthesis, thus enabling neutralization of gastric acid and promoting survival in the acidic gastric environment (de Reuse et al., 2013). These differential microbial metabolic pathways indicate that compared to that in children with clinically diagnosed *H. pylori* infection, the gastric microbiota in occult *H. pylori* infection orchestrates a coordinated response involving transcription factors and transport systems to maintain homeostasis within the “nutrient transport-gene regulation-metabolic network.” Compared to the *H. pylori*-negative group, the occult *H. pylori* infection group showed significant enrichment of genes related to cell proliferation, stress adaptation, and substance transport. This finding suggests that the microbiota in this group retains relatively high metabolic activity and proliferative capacity, actively reconstructing a more complex and competitive microbial ecosystem. For example, multiple ABC transporters contribute to *H. pylori* colonization (Davidson et al., 2008): the σ70 factor plays a key role in stress response and environmental adaptation (Sharma et al., 2009), and single-stranded DNA binding proteins are involved in DNA replication and replication metabolism modulation in spiral and coccoid forms of *H. pylori* (de Reuse et al., 2013).

The present study has certain limitations. First, ethical considerations precluded the inclusion of a healthy, asymptomatic control group, thereby limiting our ability to establish a true physiological baseline for the gastric microbiota and constraining interpretation of the direction and magnitude of dysbiosis associated with occult *H. pylori* infection. Second, as a cross-sectional study, this work did not assess key biological modifiers—including environmental exposures (e.g., diet), *H. pylori* virulence determinants (e.g., *cagA* and *vacA* genotypes), or host genetic variants—that may influence susceptibility to, establishment of, and persistence of occult infection. Third, although negative controls yielded no detectable amplicons, low-biomass gastric biopsies remain inherently susceptible to contamination from reagents or the laboratory environment. Genera such as *Ralstonia* and *Cupriavidus*—enriched in the occult infection group—have been previously reported as common contaminants in low-microbial-biomass studies (Salter et al., 2014; Eisenhofer et al., 2019). Consequently, their observed enrichment should be interpreted with caution, and any potential biological relevance must be rigorously validated using orthogonal approaches—such as culture-based isolation, quantitative PCR, or fluorescence in situ hybridization. Fourth, all molecular assays detect *H. pylori* DNA but cannot distinguish between viable, non-viable, or fragmented bacterial cells; therefore, a positive molecular result indicates the presence of *H. pylori* DNA—not necessarily active or viable colonization. Collectively, these considerations highlight the critical need for prospective, longitudinal studies with extended follow-up periods; integrated multi-omics profiling—including metagenomic, metatranscriptomic, and host genomic analyses; and clinically well-annotated endpoints. Such studies are essential to clarify the natural history of occult *H. pylori* infection and to determine whether molecular detection of the pathogen predicts subsequent clinical outcomes—including progression to overt gastritis, peptic ulcer disease, or therapeutic failure.

## Conclusion

The above findings not only confirm the presence of occult *H. pylori* infection in this pediatric cohort, but also indicate systematic alterations in both the taxonomic composition and functional potential of the gastric microbiota—changes that may facilitate the establishment and persistence of low-abundance *H. pylori*. Gastric microbiota features significantly associated with latent infection include: (i) marked enrichment of the genera Ralstonia and Bacteroides; (ii) a significant negative correlation between *Akkermansia* and *Helicobacter* abundance; and (iii) substantial enrichment of PICRUSt2-predicted functional pathways encompassing key metabolic and regulatory modules—including ABC transporter systems, environmental stress response mechanisms, and RNA polymerase σ^70^ factor activity.

Although current evidence remains insufficient to warrant the integration of molecular testing into routine clinical screening protocols, this study establishes a robust methodological foundation and articulates well-defined, testable scientific hypotheses to guide future large-scale, multicenter, longitudinal cohort studies. Such studies aim to systematically characterize the natural history, histopathological correlates, and long-term implications for gastric mucosal health in children harboring occult *H. pylori* infection. Notably, for the pediatric subgroup exhibiting repeatedly negative results on standard clinical assays—including rapid urease testing and histopathological examination—yet persistently experiencing upper gastrointestinal symptoms, the potential clinical utility of targeted molecular retesting warrants prospective validation.

## Data Availability

The raw 16S rRNA sequencing data have been deposited in the NCBI Sequence Read Archive (SRA) under BioProject accession number PRJNA1469871.

## Footnotes

1
https://qiime2.org
