# Supplementary material for: Occult Helicobacter pylori infection in children: molecular detection and association with gastric dysbiosis
Source: Front Microbiol. 2026 Jun 15;17:1836526. doi: 10.3389/fmicb.2026.1836526 (PMC13312810; doi:10.3389/fmicb.2026.1836526)
Supplement: Supplementary file 1 [file Table_1.docx]

Supplementary Material

## Supplementary Figures


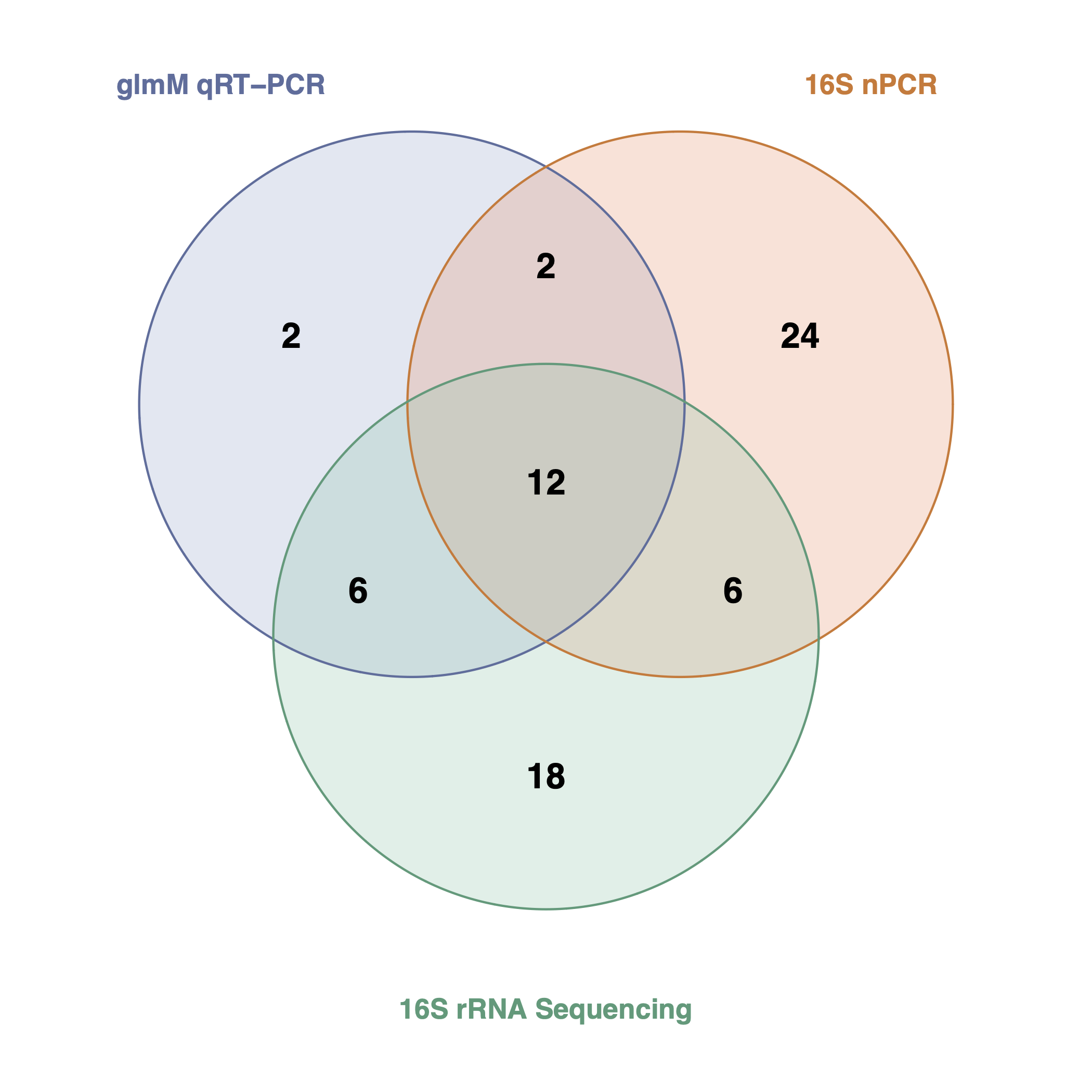


Supplementary Figure 1. Venn diagram illustrating the overlap of positive results among three molecular assays for *H. pylori* detection across all 153 enrolled children. The three methods were glmM qRT‑PCR, *H. pylori*‑specific 16S rRNA nested PCR (16S nPCR), and 16S rRNA gene sequencing (16S seq). Numbers within each exclusive and overlapping region indicate the count of positive samples. Across the entire cohort, 2 samples were positive only by glmM qRT‑PCR, 24 only by 16S nPCR, and 18 only by 16S seq. Pairwise overlaps: 2 samples were positive by both glmM qRT‑PCR and 16S seq, 6 by both 16S seq and 16S nPCR, and 6 by both glmM qRT‑PCR and 16S nPCR. A total of 12 samples were positive by all three methods. Note that the sum of all numbers in the diagram (70) is less than 153, as the remaining samples tested negative by all three molecular assays.


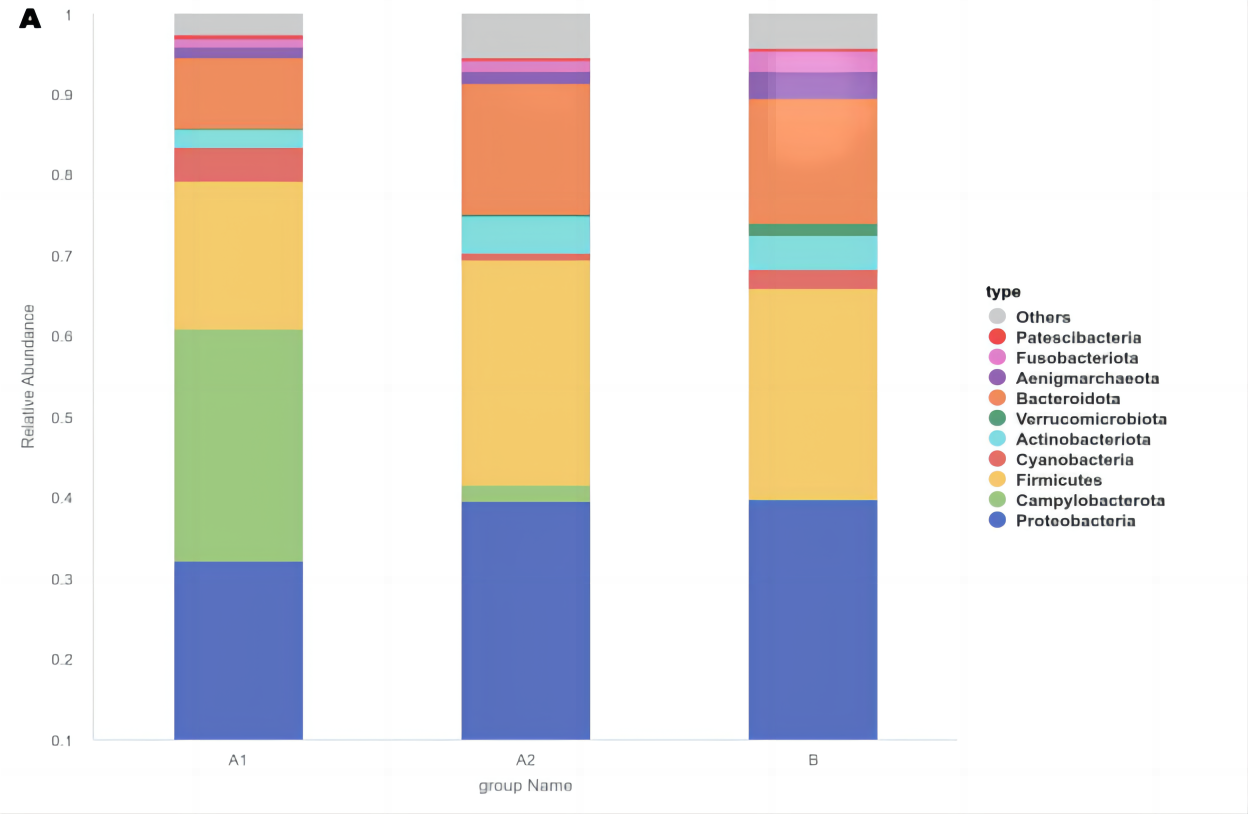


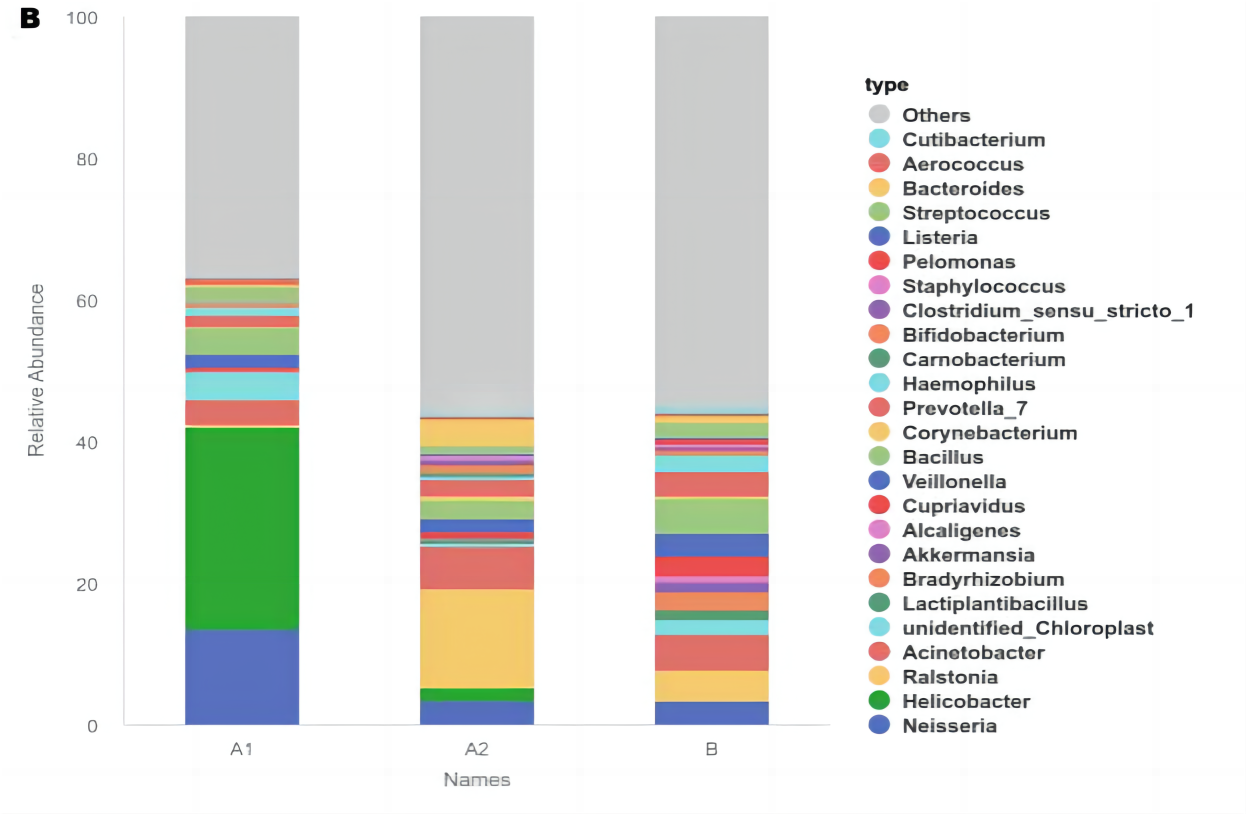


**Supplementary Figure 2.** Differences in the abundance of gastric mucosal microbiota across groups. (A) Relative abundance of dominant bacterial taxa at the phylum level. (B) Relative abundance of dominant bacterial taxa at the genus level. Abundances are expressed as proportions of total genera per subject.

Group A1: clinical diagnosed *H. pylori* infection group; Group A2: Occult *H. pylori* infection group; Group B: *H. pylori*-negative group.


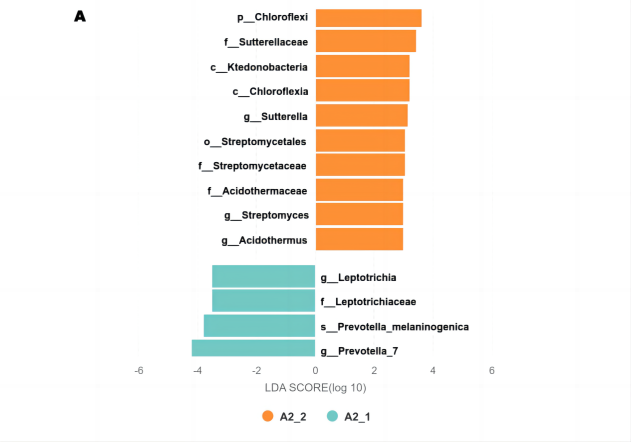

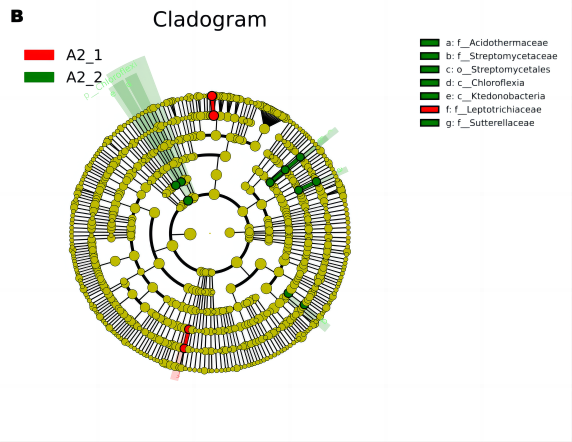


**Supplementary Figure 3.** LEfSe analysis of bacterial taxa associated with chronic inflammation severity in the occult *H. pylori* infection group. (A) Histogram of LDA scores showing differentially abundant taxa between Groups A2-1 (grade 1 chronic inflammation) and A2-2 (grade 2 chronic inflammation). (B) Cladogram illustrating the taxonomic differences between Groups A2-1 and A2-2. *Sutterella, Streptomyces,* and *Acidothermus* were enriched in patients with grade 2 chronic inflammation in the occult *H. pylori* infection group, suggesting a potential role of these genera in modulating inflammatory severity in the context of low-density *H. pylori* colonization.

Group A2-1: occult *H. pylori* infection with chronic inflammation of grade 1 group. Group A2-2: occult *H. pylori* infection with chronic inflammation of grade 2 group.


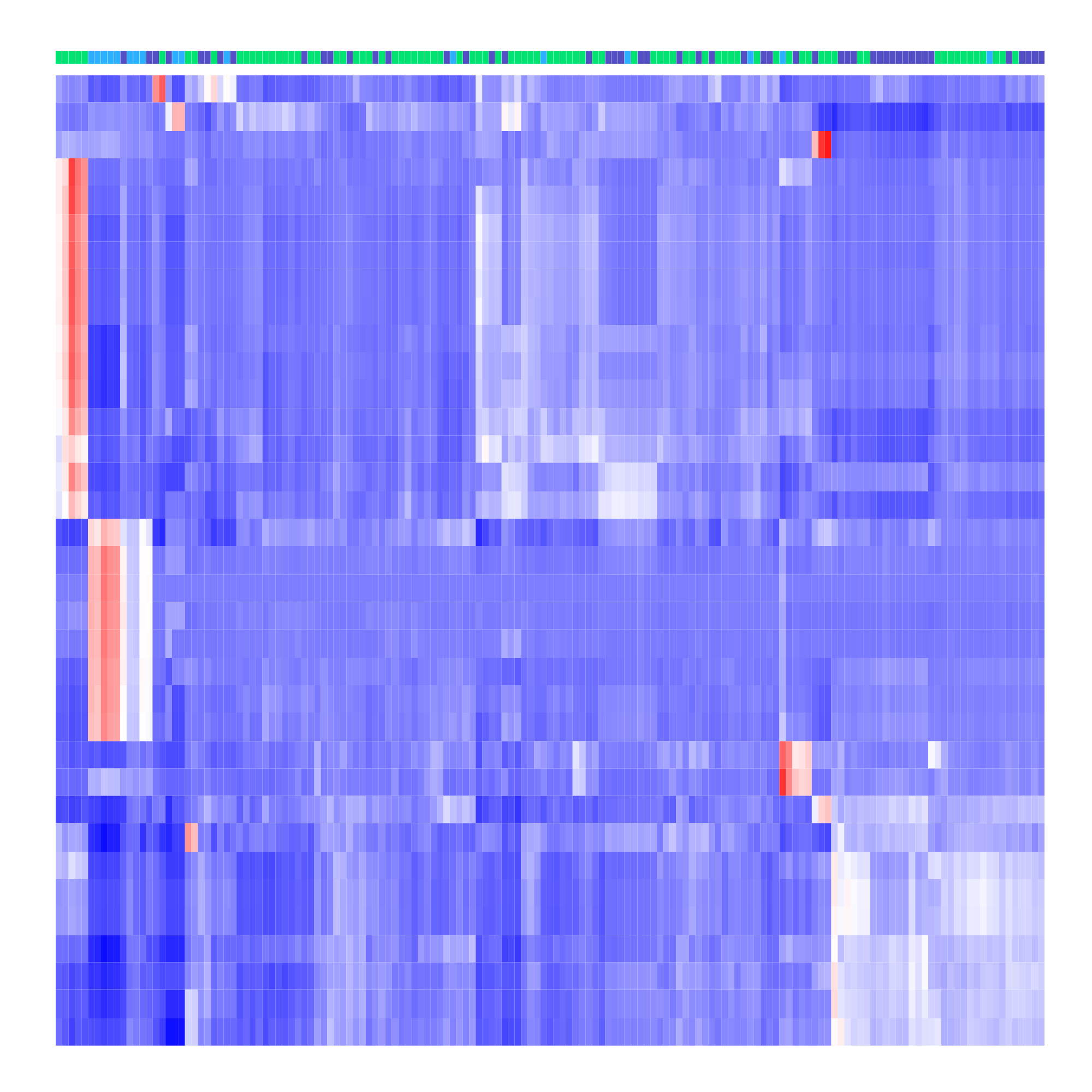


**Supplementary Figure 4.** Heatmap of predicted gastric microbiota function in KEGG pathway among three Groups. Group A1: clinical diagnosed *H. pylori* infection group; Group A2: occult *H. pylori* infection group; Group B: *H. pylori*-negative group.
